# 3'-coterminal subgenomic RNAs and putative *cis*-acting elements of *Grapevine leafroll-associated virus 3 *reveals 'unique' features of gene expression strategy in the genus *Ampelovirus*

**DOI:** 10.1186/1743-422X-7-180

**Published:** 2010-08-03

**Authors:** Sridhar Jarugula, Siddarame Gowda, William O Dawson, Rayapati A Naidu

**Affiliations:** 1 Department of Plant Pathology, Irrigated Agriculture Research and Extension Center, Washington State University, Prosser, WA 99350, USA; 2 Citrus Research and Education Center, University of Florida, Lake Alfred, FL 33850, USA

## Abstract

**Background:**

The family *Closteroviridae *comprises genera with monopartite genomes, *Closterovirus *and *Ampelovirus*, and with bipartite and tripartite genomes, *Crinivirus*. By contrast to closteroviruses in the genera *Closterovirus *and *Crinivirus*, much less is known about the molecular biology of viruses in the genus *Ampelovirus*, although they cause serious diseases in agriculturally important perennial crops like grapevines, pineapple, cherries and plums.

**Results:**

The gene expression and *cis*-acting elements of *Grapevine leafroll-associated virus 3 *(GLRaV-3; genus *Ampelovirus*) was examined and compared to that of other members of the family *Closteroviridae*. Six putative 3'-coterminal subgenomic (sg) RNAs were abundantly present in grapevine (*Vitis vinifera*) infected with GLRaV-3. The sgRNAs for coat protein (CP), p21, p20A and p20B were confirmed using gene-specific riboprobes in Northern blot analysis. The 5'-termini of sgRNAs specific to CP, p21, p20A and p20B were mapped in the 18,498 nucleotide (nt) virus genome and their leader sequences determined to be 48, 23, 95 and 125 nt, respectively. No conserved motifs were found around the transcription start site or in the leader sequence of these sgRNAs. The predicted secondary structure analysis of sequences around the start site failed to reveal any conserved motifs among the four sgRNAs. The GLRaV-3 isolate from Washington had a 737 nt long 5' nontranslated region (NTR) with a tandem repeat of 65 nt sequence and differed in sequence and predicted secondary structure with a South Africa isolate. Comparison of the dissimilar sequences of the 5'NTRs did not reveal any common predicted structures. The 3'NTR was shorter and more conserved. The lack of similarity among the *cis*-acting elements of the diverse viruses in the family *Closteroviridae *is another measure of the complexity of their evolution.

**Conclusions:**

The results indicate that transcription regulation of GLRaV-3 sgRNAs appears to be different from members of the genus *Closterovirus*. An analysis of the genome sequence confirmed that GLRaV-3 has an unusually long 5'NTR of 737 nt compared to other monopartite members of the family *Closteroviridae*, with distinct differences in the sequence and predicted secondary structure when compared to the corresponding region of the GLRaV-3 isolate from South Africa.

## Background

The family *Closteroviridae *comprises genera with monopartite genomes, *Closterovirus *and *Ampelovirus*, and with bipartite and tripartite genomes, *Crinivirus *[1]. They are semi-persistently transmitted by aphids (closteroviruses), whiteflies (criniviruses) or mealybugs/scale insects (ampeloviruses) and represent the most complex plant viruses infecting a broad range of agriculturally important crops [2]. Closteroviruses in the genera *Closterovirus *and *Crinivirus *have complex genome organizations and expression strategies unique to the viruses in the family *Closteroviridae *[[3-12] and citations in these references]. The unusually long, highly flexuous filamentous particles have bipolar architecture composed of at least two capsid proteins which encapsidate single-stranded, positive-sense RNA genomes of ~15-20 kb [7,8]. The replication-associated proteins are encoded by a signature 'replication gene block', made up of domains for one or two papain-like proteinases, methyl transferase- and helicase-like domains with large interdomain region, and a +1 frameshift to express an RNA-dependent RNA polymerase-like domain. The other genes are encoded in 7-12 open reading frames (ORFs) and are expressed through a nested set of 3'-coterminal subgenomic (sg) mRNAs. Among these genes is a signature 'quintuple gene module' involved largely in assembly of virions. The other ORFs vary in number and arrangement and appear to be unique to each species in the family.

Based on the well-studied closteroviruses and criniviruses, the different 3' genes are expressed at greatly variable amounts, suggesting precise regulation of different proteins in relation to the amounts needed during the virus life cycle. With *Citrus tristeza virus *(CTV) as a model, there appear to be general rules that determine the levels of production of the different 3'-coterminal sgRNAs. First, genes located nearer to the 3' terminus tend to be expressed at higher levels than internal genes. The second rule is that ORFs with an upstream nontranslated region are generally expressed higher than those ORFs that overlap or do not have an upstream nontranslated region. With CTV, the *cis*-acting elements that regulate the level of expression of genes in the 3' half of the genome are located immediately upstream to the transcription start site of their sgRNAs. These elements generally consist of one or two stem-loop (SL) structures with a downstream (plus sense) +1 site corresponding to the 5' terminal adenosine of the sgRNA [13,14]. Additionally, an adenylate appears to be the 5'-terminus of all sgRNAs encoded by CTV similar to the 5'terminus of the genomic RNA [15]. In the case of *Beet yellows virus *(BYV), several sgRNAs have adenylate at their 5'termini, with the exception of BYV p6 sgRNA that contains a guanylate similar to the 5' terminus of the genomic RNA [16,17]. On the other hand, the 5'terminal nucleotide of the sgRNAs of the crinivirus *Sweet potato chlorotic stunt virus *was reported to be variable, having adenylate, guanylate or uridylate, and the 5' ends of genomic RNA 1 and RNA 2 have conserved guanylates [18].

By contrast, much less is known about the molecular biology of closteroviruses in the genus *Ampelovirus*, although they cause serious diseases in agriculturally important perennial crops like grapevines [19], pineapple [20], cherries [21] and plums [22]. *Grapevine leafroll-associated virus 3 *(GLRaV-3), the type member of the genus *Ampelovirus*, represents the second largest virus in the family *Closteroviridae *with a monopartite genome of 18,498 nt [23], after CTV that has a 19,293 nt genome [24]. Similar to CTV, molecular variants of GLRaV-3 have been documented using partial [25] and full length sequences [23,26,27]. An analysis of the sequences of GLRaV-3 isolates showed similar genome organization with a relatively high degree of nucleotide conservation across their genome, except in the 5' nontranslated region (NTR). Also, the length of the 5'NTR was reported to be different for different isolates. The South Africa isolate was reported to have a 737 nt long 5'NTR [23], whereas New York [26] and Chile [27] isolates were reported to have 158 nt 5'NTRs.

**Figure 1:**
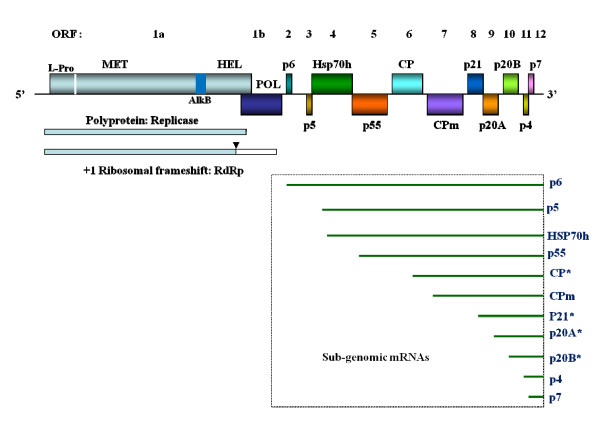
**A schematic diagram of the GLRaV-3 genome**. The ORFs, numbered as 1 to 12 above the diagram, are shown as boxes with associated protein designations. L-Pro, leader proteinase; AlkB, AlkB domain; MET, HEL, and POL, methyltransferase, RNA helicase, and RNA-dependent RNA polymerase domains of the replicase, respectively; p6, a 6-kDa protein; p5, a 5-kDa protein; HSP70 h, a HSP70-homologue; p55, a 55-kDa protein; CP, the major capsid protein; CPm, the minor capsid protein; and p21, p20A, p20B, p4 and p7 are the 21-, 19.6-, 19.7-, 4- and 7-kDa proteins, respectively. Below the genome map is a representation of (right) the 11 putative subgenomic messenger (m) RNAs for the 3' genes and (left) the polyproteins from ORFs 1a and 1b. The subgenomic mRNAs and their transcription start sites identified in this study are shown with an asterisk. Arrow head indicates site of +1 ribosomal frameshift.

The genome organization of GLRaV-3 is shown in Fig. 1. Unlike other viruses in the genera *Closterovirus *and *Ampelovirus*, GLRaV-3 contains two small ORFs (p7 and p4) nearest to the 3'-terminus of the genome. In the case of BYV and CTV, the most 3'-proximal ORFs encode highly expressed ~p21 kDa and ~p23 kDa proteins, respectively, that function as replication enhancers [28,29]. In GLRaV-3, p20B, the counterpart to the BYV p21 ORF or CTV p23 ORF, is present upstream of p7 and p4. Thus, it appears that p7 and p4 are unique to GLRaV-3 and counterparts of these genes are not present in other closteroviruses. Additionally, the order of arrangement of CP and CPm is different in GLRaV-3 with the latter located towards the 3'-terminus of the virus genome, when compared to their arrangement in viruses of the genus *Closteroviruses*. Moreover, the size of CPm of GLRaV-3 is much larger than that of BYV and CTV.

In this study, we examined the gene expression strategy and *cis*-acting elements of GLRaV-3 in comparison to the other members of the *Closteroviridae*. Four of the eleven putative 3'-coterminal sgRNAs accumulated at high levels, two at intermediate levels, and the rest at low levels in naturally infected grapevine tissues. The transcription start sites of the four abundantly expressed sgRNAs were determined relative to the genomic RNA and their leader sequences and upstream sequences, where *cis*-acting sequences would be expected, were analyzed as a first step to elucidate gene expression strategy in ampeloviruses. The results indicate that transcription regulation of GLRaV-3 sgRNAs appears to be different from members of the genus *Closterovirus*. An analysis of the genome sequence confirmed that GLRaV-3 has an unusually long 5'NTR of 737 nt compared to other monopartite members of the family *Closteroviridae*, with distinct differences in the sequence and predicted secondary structure when compared to the corresponding region of the GLRaV-3 isolate from South Africa. In contrast, the 3'NTR of the two isolates is highly conserved.

## Results

### Some 3'-coterminal sgRNAs are abundantly present in grapevines naturally infected with GLRaV-3

**Figure 2:**
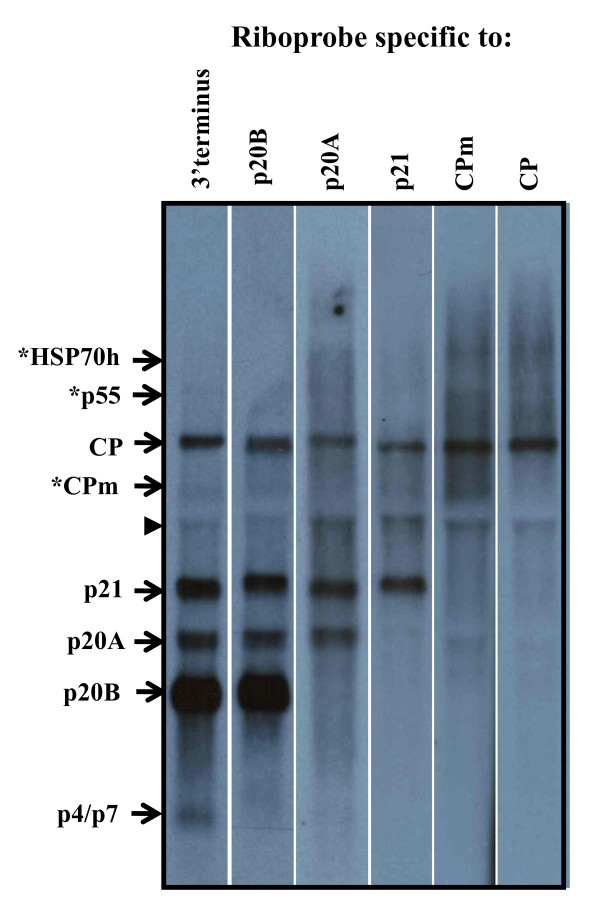
**Northern blot analysis of total RNA extracted from grapevine (cv. Merlot) infected with GLRaV-3**. Northern blot hybridizations were carried out using a positive-stranded gene-specific riboprobes containing 3'terminus, p20A, p21, CPm, and CP sequences. Position of subgenomic (sg) RNAs is indicated by arrows on the left. Location of sgRNAs for CPm, p55 and HSP70h were tentative and indicated with an asterisk. The non-specific band present in all lanes is indicated by an arrow head.

By analogy with BYV [4] and CTV [6], the two well studied members of the genus *Closterovirus*, ORFs 2 through 12, covering the 3' half of the GLRaV-3 genome (Fig. 1) would be expected to be expressed via eleven 3'-coterminal sgRNAs. As a first step towards comparative exploration of replication strategy of viruses in the genus *Ampelovirus*, we investigated the presence of sgRNAs in grapevine naturally infected with GLRaV-3. Total RNA preparations from scrapings of bark tissues were analyzed by Northern blot hybridization with positive-stranded RNA-specific riboprobes corresponding to nts 17,899 to 18,498 at the 3' end of GLRaV-3 genomic RNA. As shown in Fig. 2, four sgRNAs were present at higher levels and they were putatively identified as specific to p20B (ORF 10), p20A (ORF 9), p21 (ORF 8) and CP (ORF 6) genes. The two sgRNAs for p4 (ORF11) and p7 (ORF12) were not resolved due to the small differences in their sizes and appeared as a single moderately expressed band in Northern blots. The three barely visible bands were putatively identified as sgRNAs corresponding to CPm (ORF 7), p55 (ORF 5) and p5+HSP70 h (ORFs 3 and 4) genes. The specificity of the abundantly-accumulated sgRNAs to CP, p21, p20A and p20B genes was further confirmed by hybridization with riboprobes prepared using gene-specific sequences (Fig. 2). The riboprobe specific to the CPm hybridized weakly with the corresponding sgRNA band. Since the riboprobe showed strong hybridization with sgRNA of the CP, the observed weak signal further confirms that the sgRNA of CPm is poorly expressed. Among the four sgRNAs that accumulated at higher levels, the sgRNA corresponding to p20B gene accumulated at the highest level, followed by sgRNAs for p21, p20A and CP, respectively (Fig. 2). These results suggest that 3'-coterminal sgRNAs accumulate at variable amounts, reflecting differences in their expression levels and/or turnover rates in infected grapevine tissues. None of the sgRNAs were detected with a riboprobe specific to the 5'NTR (data not shown) further confirming that they are 3'-coterminal to the virus genome.

### GLRaV-3 has an unusually long 5'NTR

In order to characterize the sgRNAs further and map their locations in the virus genome, we needed to obtain the sequence of the Washington isolate of GLRaV-3. Although sequence is available for South Africa, Chile and New York isolates of GLRaV-3, considerable variation in their genome size between 17,919 and 18,498 nt warranted generating full genome sequence of the Washington isolate. In addition, having genome sequence information for the parental isolate of GLRaV-3 would enable mapping the 5'-transcription start site of sgRNAs more precisely in the cognate viral genome sequence. Due to its large size, the entire genome of GLRaV-3 was amplified into seven segments using virus-specific primers (Additional file 1, Figure S1 and Table S1). The cDNA clones representing each of the genomic segments were sequenced by directed sequencing protocol ("DNA walking") using progressive sequence-specific primers designed based on the partial nucleotide sequence obtained. This strategy, instead of cloning and sequencing by random oligonucleotide primers, decreased the number of steps required for determining the complete genome sequence of the virus and assembling the consensus sequence into a full-length genomic RNA sequence.

The RNA genome sequence of Washington isolate of GLRaV-3 was determined to be 18,498 nt long and it was deposited in GenBank under the accession no. GU983863. The genome contains thirteen putative ORFs with 737 nt long 5'NTR and 277 nt long 3'NTR (Fig. 1). The genome organization of Washington isolate was identical to GLRaV-3 isolates from New York [26], Chile [27] and South Africa [23]. The sizes of different ORFs and the 3'NTR were similar between all isolates (Additional file 1, Table S2). However, the size of the 5'NTR was significantly different, with New York and Chile isolates containing 158 nt, and South Africa and Washington isolates having 737 nt. In general, the genome of Washington isolate of GLRaV-3, downstream of 5'NTR sequence, showed higher level of nucleotide sequence identity with corresponding sequence of virus isolates from New York (~97%) and Chile (~99%) than with South Africa isolate (~92%). Overall, higher sequence identity values indicate that Washington isolate is closely related to GLRaV-3 isolates from New York and Chile than to the South Africa isolate (Additional file 1, Table S2).

**Figure 3:**
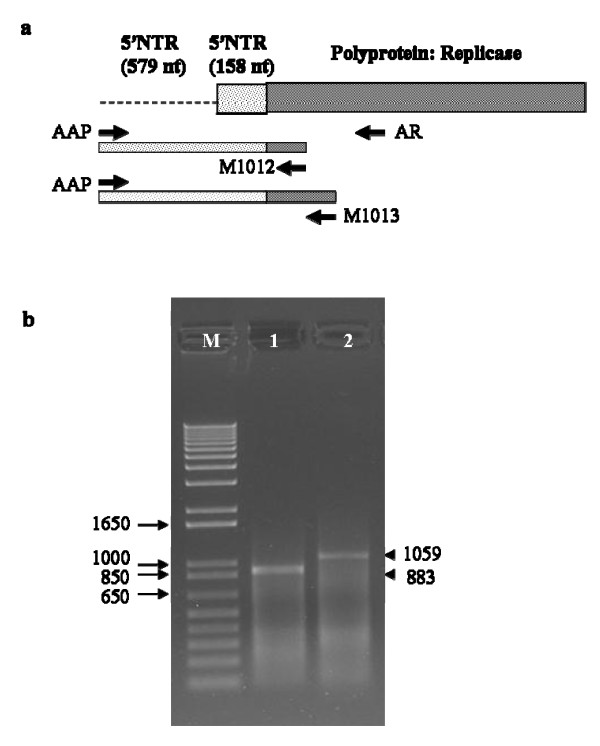
**RACE analysis of 5'NTR of GLRaV-3**. (a) The schematic diagram showing the locations of primers used and expected size of amplicons and (b) agarose gel showing virus-specific DNA fragments (shown by arrow head on the right) amplified from cDNA made using primer AR. Lane 1 shows 883 bp fragment amplified with primers AAP and M1012 and lane 2 shows 1059 bp fragment amplified with primers AAP and M1013 primers. Lane M shows 1kb plus DNA marker (Invitrogen) for estimating the size of amplified DNA fragment. The size of marker DNA bands is indicated to the left. See Materials and methods for primer details.

Due to the discrepancy in the size of 5'NTR of the four GLRaV-3 isolates, we examined the sequence of 5'NTR of several isolates from six cultivars: four wine grape cultivars (Cabernet Sauvignon, Syrah, Merlot, Chardonnay), one table grape cultivar (Thomson Seedless) and one juice grape cultivar (Concord) planted in geographically widely separated regions in the US. The 5'RACE system was employed to verify the exact size of 5'NTR using two gene-specific downstream primers complementary to 860 to 883 nt (primer M1012) and 1034 to 1059 nt (primer M1013) of ORF1a of the Washington isolate. The expected DNA fragments from RT-PCR amplification would be ~304 nt and ~480 nt, if the 5'NTR is 158 nt in size as reported in New York and Chile isolates or it would be ~883 nt and ~1059 nt, if the 5'NTR is 737 nt in size as found in isolates from Washington and South Africa (Fig. 3a). Using the abridged anchor primer supplied with the 5'RACE kit as an upstream primer (primer AAP), a single product of ~883 bp and ~1059 bp were amplified with virus-specific primers M1012 and M1013, respectively (Fig. 3b). Sequence analysis of eight independent clones for each isolate showed that the size of 5'NTR is 737 nt with 98-100% sequence identity with corresponding sequence of the Washington isolate. The 5'NTR is A-U rich (22.12% As and 47.49% Us) and showed 83% nucleotide identity with the 5'NTR of the South Africa isolate. The 158 nt 5'NTR sequence of the Washington isolate immediately upstream of ORF1a showed 100% identity with corresponding 5'NTR sequences of New York and Chile isolates. From these results it is clear that the 737 nt 5'NTR is indeed authentic and an unusually long nontranslated sequence could be characteristic of GLRaV-3 including New York and Chile isolates.

### The 5'NTR of GLRaV-3 isolates shows complex but distinct structural architecture than 3'NTR

**Figure 4:**
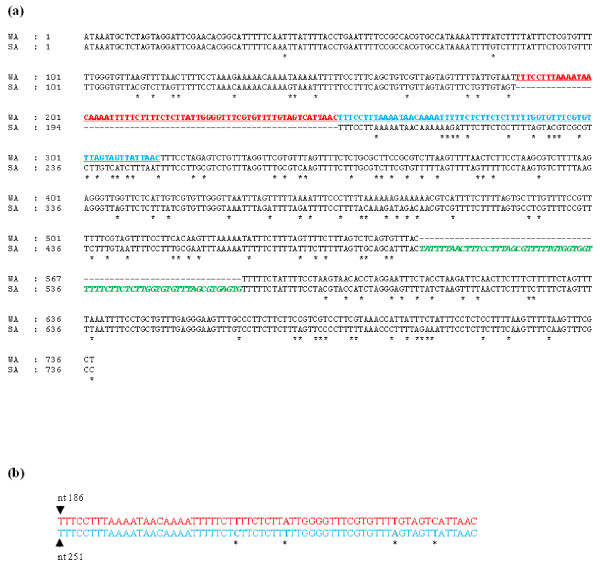
**Pairwise comparison of 5'NTR sequences of GLRaV-3 isolates from Washington (WA) and South Africa (SA)**. (a) The alignment was adjusted manually and residues that are unique to each isolate are shown by an *asterisk *beneath them. The 65 nt tandem repeat in WA isolate is represented in bold and as underline (red and blue colored text), and extra sequence present only in South Africa isolate is in bold italics (green colored text), (b) pairwise alignment of 65 nt tandem repeat in Washington isolate showing differences at four nucleotide positions.

**Figure 5:**
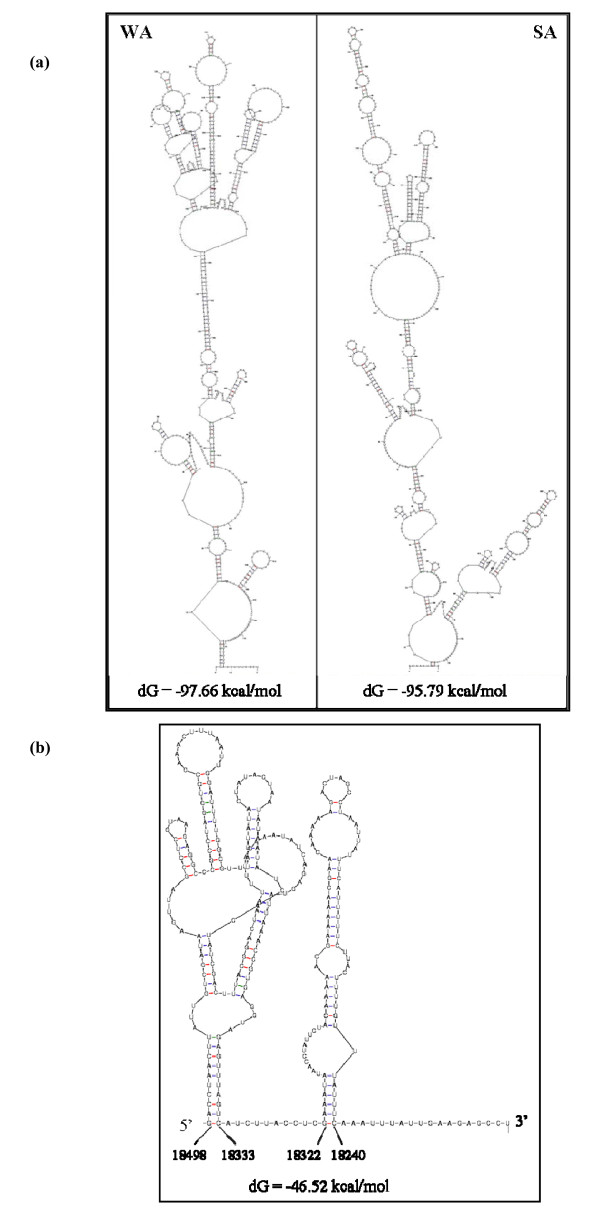
**The computer-predicted secondary structure of the NTRs of GLRaV-3**. (a) 5'NTR of Washington (WA) and South Africa (SA) isolates and (b) 3'NTR of WA isolate (complement).

Although the 5'NTRs of several GLRaV-3 isolates from the US and South Africa were of the same size, pairwise comparison showed non-uniform sequence identity distributed across the entire sequence (Fig. 4a). An unusually long stretch of 65 nt tandem repeat was observed between nucleotides 187 to 315 in the 5'NTR of all isolates of GLRaV-3 from the US sequenced in this work, where the first repeat was found between nucleotides 187-250 and the second between 251-315. Four nucleotide differences were observed in the tandem repeat sequences and these differences were distributed randomly in the entire length of the repeat (Fig. 4b). Whereas, such a signature tandem repeat was absent in 5'NTR of the South Africa isolate. However, the South Africa isolate has an additional 65 nt sequence that maintained 737 nt size of its 5'NTR (Fig. 4a). Alignment of 5'NTR sequences showed high sequence identity in the end sequences and in the middle portion with two distinct, highly variable regions in between. To further examine differences in the 5'NTR of GLRaV-3 isolates, we compared their predicted secondary structure using computational calculations at the MFOLD web server [30]. The 5'NTR of Washington and South Africa isolates folded into a complex structure consisting of a long SL structure with several substructural hairpins of variable lengths (Fig. 5a). This indicated that, although both isolates of GLRaV-3 have similar size 5'NTRs, the primary sequence and the predicted secondary structural architecture differed between them. In contrast, the 277 nt 3'NTR of Washington, New York, Chile and South Africa isolates of GLRaV-3 showed >97% similarity and folded into identical secondary structures (complementary sequence) consisting of two long SL structures (Fig. 5b). The 5' most SL consisted of 166 nt (18,333-18,498) and the 3'most structure with 83 nt (18,240-18,322) with the 5' most one forming a complex structure containing four substructural hairpins of variable length.

### Mapping the transcription start sites of the most abundant sgRNAs revealed differences with other monopartite members of the family *Closteroviridae*

**Figure 6:**
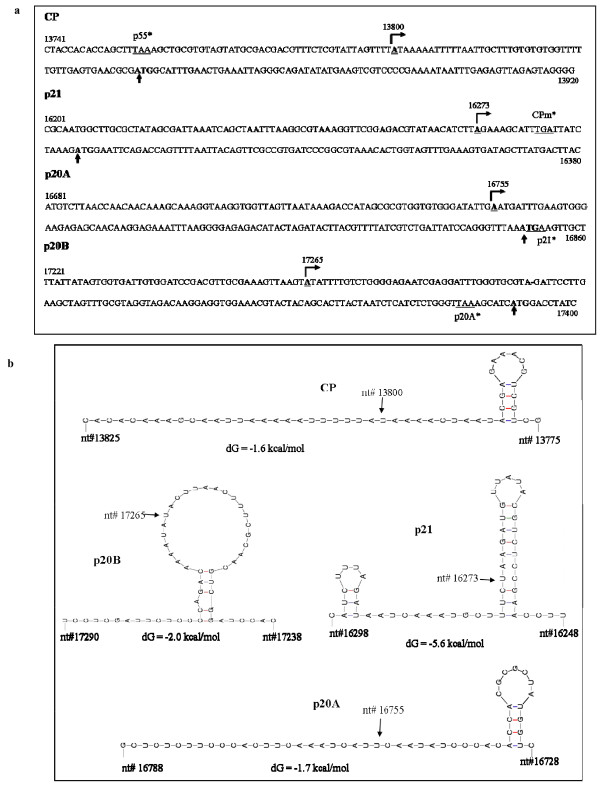
**Transcription start site (TSS) of four subgenomic (sg) RNAs**. (a) Nucleotide sequence of the portion of the genomic RNA showing the TSS of sgRNAs specific to CP, p21, p20A and p20B and (b) the predicted secondary structure of the minus-strand sequences around the TSS of the sgRNAs. Numbers indicate nucleotide coordinates with the genomic RNA. The TSS is indicated by a bent arrow (with +1 adenylate underlined) in (a) and by an arrow in (b), termination codon of the preceding ORF is underlined and marked with an asterisk and the translation initiation codon is in bold and marked with an arrow.

The members of the genera *Closterovirus *and *Crinivirus *employ the production of sgRNAs to serve as messengers for specific genes as one of the adaptive strategies to express their polycistronic genomes in their hosts [4-6]. As a first step toward understanding strategies underlying production of the sgRNAs of viruses in the genus *Ampelovirus*, we mapped the 5' termini of the four most abundantly expressed genes in relation to the genomic RNA of GLRaV-3. For this purpose, the 5' ends of CP, p21, p20A and p20B sgRNAs were RT-PCR amplified by the 5'RACE system from total RNA isolated from grapevine tissue infected with GLRaV-3 using a combination of an abridged anchor primer and gene-specific primer, and the amplicons were cloned into pGEM-T vector. Sequences obtained from six independent clones for each of the sgRNAs were identical and were subsequently used to map the exact nucleotide position of the 5'-end of each sgRNA. The results showed that the length of sequence between the 5'-end and the putative start codon of each ORF (sgRNA leader sequence) is 48, 23, 95 and 125 nt, respectively, for CP, p21, p20A and p20B sgRNAs (Fig. 6a). This data demonstrated that the four sgRNAs have distinctly different sizes of mRNA leader sequences that are collinear with the genomic RNA. All four sgRNAs started with an adenylate, similar to the 5'-end of the genomic RNA. Based on this information, the transcription start site (TSS) for CP, p21, p20A and p20B was located at 13,800, 16,273, 16,755 and 17,265 nt, respectively, in the genome sequence of the Washington isolate of GLRaV-3 (Additional file 1, Table S3). The 48 nt leader sequence of CP sgRNA is located entirely in the intergenic region (IGR) between p55 and CP, the 23 nt leader sequence of p21 encompass 13 nt C-terminus of CPm ORF and 10 nt IGR between CPm and p21, the 95 nt leader sequence of p20A overlaps with the C-terminus of p21, and the 125 nt leader sequence of p20B encompass 119 nt C-terminal portion of p20A and 6 nt IGR between p20A and p20B. Using the location of TSS, we estimated the size of sgRNA for CP, p21, p20A and p20B (Fig. 6a) as 4,699, 2,226, 1,744 and 1,234 nts, respectively (Additional file 1, Table S3). The TSS for CP, p20A and p20B sgRNAs match with those reported for South African isolate of GLRaV-3 [31]. The study by Maree *et al. *[31] also indentified TSS for the other 3' sgRNAs. However, we could not amplify 5'-end sequences for the other sgRNAs (ORFs 2, 3, 4, 5, and 7), despite several attempts, possibly due to their low abundance in infected grapevine tissue.

The 5' leader sequences of CP, p21, p20A and p20B of the Washington isolate were more similar (98-100% identity) to the corresponding sequences in the New York and Chile isolates than with South Africa isolate (88-94% identity) (Additional file 1, Table S4). Except for the 5'-end nucleotide, the leader sequences of the four sgRNAs did not reveal any shared sequence motifs, in contrast with the presence of a conserved heptanucleotide in some sgRNAs of BYV and CTV [16]. Since conserved nucleotide sequences around the TSS of sgRNA were implicated in the synthesis of sgRNAs of *Tobacco mosaic virus *(TMV) [32] and *Citrus tatter leaf virus *(CTLV) [33], we compared a 50 nt region around the TSS (-25 to +25 relative to the initiation site of each sgRNA) of each of the four sgRNAs of GLRaV-3. The results revealed no conserved sequences surrounding the TSS, in contrast to the presence of conserved octanucleotide sequences in TMV and CTLV.

The sequences upstream of the ORFs of CTV were shown to control production of sgRNAs [13,14]. In general, the minus strands corresponding to these regions could be folded into one or two SL structures with the first nucleotide (A) of the sgRNA leader a few nucleotides downstream of the last SL structure [34]. To predict the possible involvement of RNA secondary structures in the sgRNA synthesis analogous to TMV, CTLV and CTV, the 50 nt sequence corresponding to the minus strand sequence around the TSS of the four sgRNAs was analyzed by MFOLD [30]. The results showed that, although the 50 nt sequence corresponding to the minus strand sequence of sgRNAs for CP, p21, p20A and p20B folded into predicted SL structures, conservation of the predicted secondary structures was not observed (Fig. 6b). The 5'end nucleotide of sgRNA specific to CP and p20A was located outside the SL structure, whereas that specific to p21 sgRNA was located on the stem of the SL structure and it was in the loop in the case of p20B sgRNA. The integrity of these secondary structural features were maintained in the leader sequences of all GLRaV-3 isolates, despite low (84-94%) sequence identity especially between Washington and South Africa isolates, suggesting a requirement for the putative secondary structure in sgRNA synthesis. The genome sequences immediately ~100 nt upstream from the 5' terminus of each sgRNA were also compared to verify the possible conservation in the putative control elements for sgRNA production. The data revealed no similarity between the sequences or their predicted structures and no structures similar to those found for CTV.

## Discussion

Synthesis of 3'-coterminal sgRNAs is one of the genome expression strategies adapted by members of the family *Closteroviridae*. Evidence gathered with viruses in the genera *Closterovirus *[4,6] and *Crinivirus *[5,18,35] demonstrated that the temporal expression and kinetics of accumulation of these sgRNAs is highly regulated and level of sgRNAs expressed depends on promoter strength and position within the genome. However, expression strategies of sgRNAs for members of the genus *Ampelovirus *have not been studied to date, despite their economic importance to many agriculturally important crops. The characteristic profiles of sgRNAs obtained in Northern blots from virus-infected grapevine (Fig. 2) provided evidence that GLRaV-3 likely employs a strategy for the expression of its nested set of 3'-coterminal sgRNAs similar to other closteroviruses. However, accumulation of sgRNAs detected in virus-infected grapevine tissue indicated differences in the levels of expression of specific sgRNAs compared to other monopartite closteroviruses like BYV and CTV [15,16,36,37]. Since results of this study were from an asynchronous infection, it is possible that the amounts and timing of synthesis of sgRNAs in GLRaV-3-infected tissue are variable during the growing season and remains to be elucidated to determine whether their profile changes in relation to the developmental stage or expression of grapevine leafroll disease symptoms [19].

The sgRNAs for p7 and p4 ORFs, located close to the 3' terminus of GLRaV-3 genome, accumulated at lower levels than sgRNAs specific to upstream ORFs p20B, p20A and p21. Similarly, the sgRNA specific to the CP showed higher accumulation than the sgRNA specific to CPm, which is closer to the 3' terminus. Another unique feature of GLRaV-3 is that the sgRNAs specific to p21 ORF, which has only a 10 nt 5'nontranslated region upstream of the ORF where a *cis*-acting promoter element would be expected to occur, and the sgRNA corresponding to the p20A ORF, which lacks an upstream nontranslated region due to overlap with the upstream p21 ORF, accumulate at more or less similar levels compared to sgRNA of the CP ORF that has an 89 nt upstream nontranslated region. These results clearly suggest that GLRaV-3 does not follow either of the two general rules: (i) genes located nearer the 3'terminus are usually expressed at higher levels and (ii) ORFs with a 5'nontranslated region are generally expressed higher than ORFs that overlap with the upstream ORF (and hence no nontranslated region). Instead, it is likely that production of GLRaV-3 sgRNAs follows an alternative or modified mechanism.

The mapping of 5' termini of four 3'-terminal genes (CP, p21, p20A and p20B) of GLRaV-3 indicated that they possess a +1 adenylate, same as the 5' end of the genome, and this observation was analogous to CTV than to other closteroviruses like BYV [16-18]. The leader sequences of GLRaV-3 sgRNAs are collinear with the genomic RNA and their relative lengths are within the range observed for sgRNAs of CTV and BYV. The first few nucleotides of the 5' end of the genomic RNA (ATAAATG) and the genomic sequence around the 5' termini of sgRNAs (underlined, CP [TTTTATAAA], p21 [TCTTAGAAA], p20A [ATTGAATGA], p20B [AAGTATATT]) were similar but not identical AU-rich regions. Presence of an adenylate as the 5' terminus in the genomic and all four sgRNAs suggested that initiation of RNA synthesis from an uridylate on the minus strand is preferred by the GLRaV-3 replicase complex, similar to that proposed for CTV [15]. However, the lack of sequence conservation around the 5' termini and the absence of secondary structure elements or octanucleotides conserved in BYV and CTV nontranslated leader regions suggest that sgRNA expression strategy in GLRaV-3 could be somewhat different leading to the hypothesis that some commonalities do exist in the replication strategy between closteroviruses and ampeloviruses, but also some features characteristic to ampeloviruses.

In this study, the complete genomic RNA sequence of GLRaV-3 was determined to consist of 18,498 nt and is in agreement with the size of GLRaV-3 isolate reported from South Africa [23]. The overall genome organization of these two isolates is similar to that of GLRaV-3 isolates sequenced from New York and Chile. All four isolates have highly conserved 3'NTRs than 5'NTRs due to the unusual variation in size and sequence between them. Given the same size 5'NTR in two GLRaV-3 genome sequences obtained by independent groups in distant locations [this study and 23], it is likely that artifacts in cloning would have resulted in the apparent small size 5'NTR reported for New York and Chile isolates. It could be possible that use of poly (A) tailing to amplify the 5'NTR and the likelihood of non-specific annealing of oligo (dT) primer to a portion of 5'NTR with high adenines in the complementary DNA strand of GLRaV-3 would have contributed to the amplification of less-than full size sequence at the 5' terminal portion of the genome [26]. The 5'portion of GLRaV-3 isolate from Chile was amplified by RT-PCR using primers designed based on the sequence of New York isolate [27] and hence may not be a true representation of the authentic 5'end of virus genome. In contrast, our study and that reported from South Africa [23] used 5'RACE to accurately map the 5'end of GLRaV-3 genome. Additionally, same size PCR products with high level of sequence similarity amplified from GLRaV-3 isolates originating from different grapevine cultivars planted in geographically distinct locations in the US provided additional evidence that the size of 5'NTR determined in our study is accurate.

A 737 nt long 5'NTR in GLRaV-3 genome represents the longest 5'NTR among the currently known monopartite members of the family *Closteroviridae *available in database. The sizes of 5'NTR in viruses of the genus *Closterovirus *is between 105 and 227 nt and those in the genus *Ampelovirus *between 213 and 737 nt, respectively. In contrast, the bipartite and tripartite members of the genus *Crinivirus *have 72 to 264 nt 5'NTR in RNA 1. Thus, highly variable size of 5'NTR appears to be a characteristic of the members in the genus *Ampelovirus *in the family *Closteroviridae*. In addition, the 5'NTRs of grapevine-infecting closteroviruses, such as GLRaV-1, GLRaV-2 and GLRaV-Pr, for which complete genome sequences are available in the database, are variable in size with no significant sequence homology between them. Outside of the family *Closteroviridae*, a long 5'NTR of 739 nt was recently observed in *Triticum mosaic virus *(TriMV), a wheat-infecting virus in the family *Potyviridae *[38]. To our knowledge, GLRaV-3 and TriMV appears to be the only plant viruses with monopartite genome known to date with such a long sequence in their 5'NTR. A long 5'NTR in plant viruses is unusual, but a long 5'NTR ranging in length from 610 to 1500 nt has been reported in animal/human-infecting viruses in the family *Picornaviridae *[39]. Unlike 5'NTRs of TriMV and picornaviruses, which contain multiple non-conserved AUG upstream of the initiation codon, the 737 nt long 5'NTR of GLRaV-3 isolates contain only one AUG triplet at the very 5'terminus (5'-ATAAATGCTC) preceding the translation initiation codon of the ORF1 polyprotein. Thus, the long 5'NTR of GLRaV-3 appears to have features distinct from other plant and animal infecting viruses. The 5'NTR sequences in many picornaviruses and flaviviruses are highly structured to form internal ribosome entry (IRES) elements that play a role in protein translation in a cap-independent manner [40]. It remains to be studied if any potential IRES elements exist in the 5'NTR of GLRaV-3. At a practical level, the high variability in 5'NTR could be useful for discriminating GLRaV-3 isolates from different grape-growing areas around the world into phylogenetically distinct lineages.

In the case of CTV, the 5'NTRs of all isolates could be folded into two similar stem-loop structures despite the fact that their sequence varied by as much as 58% [41]. These two conserved structures were shown to be important for replication and assembly [7,42]. Even though we showed a complex secondary structure for 5'NTR of GLRaV-3 isolates (Fig. 5a) as predicted by using the MFOLD program, the complex structures did not seem to be conserved between the Washington and South Africa isolates. A tandem repeat of 65 nt in the 5'NTR sequence of many GLRaV-3 isolates from the US, but not in the 5'NTR of the South Africa isolate, is surprising and the functional importance of this repeat sequence in virus life cycle needs to be examined. In contrast to the 5'NTR, the 3'NTR of the four GLRaV-3 isolates is highly conserved and folded into identical secondary structure (Fig. 5b). This pattern is analogous to CTV, but different from BYV, where different isolates were shown to have considerable differences in size and sequence identity in the 3'NTR [28]. It has been shown with CTV that the 3'NTR contains *cis*-acting elements required to initiate synthesis of complementary negative strands and some of the predicted secondary structures are important components for efficient replication [43] and it is likely that the predicted SL structures in the 3'NTR contain *cis*-acting elements to play a critical role in GLRaV-3 replication.

## Conclusions

This study has shown that four of the eleven putative 3'-coterminal sgRNAs were abundantly present in the total RNA extracted from grapevine naturally infected with GLRaV-3 in Northern blots using gene-specific riboprobes. The 5' termini of sgRNAs specific to CP, p21, p20A and p20B were mapped in the virus genome and their leader sequences determined to be 48, 23, 95, and 125 nt, respectively, and they were collinear with the genomic RNA. The lack of conserved motifs around the transcription start site or in the leader sequences of these sgRNAs indicated that transcription regulation of GLRaV-3 sgRNAs could be different from members of the genus *Closterovirus *and suggests the lack of evolutionary conservation in the regulation of gene transcription among monopartite members of the family *Closteroviridae*. The 5'NTR of GLRaV-3 has an unusually long 5'NTR of 737 nt compared to other members of the family *Closteroviridae *and showed distinct differences in the sequence and predicted secondary structure between two distinct isolates of GLRaV-3. Availability of an infectious cDNA clone of GLRaV-3 would clearly help to explore the mechanism(s) by which the 3'-coterminal sg-mRNAs are produced and make comparative assessment of replication strategies among members of the genera *Closterovirus *and *Ampelovirus *and their adaptation to disparate plant hosts and insect vectors.

## Methods

### Virus source and preparation of double-stranded RNA

GLRaV-3 isolates were collected from five wine grape cultivars (*Vitis vinifera*, cvs. Cabernet Sauvignon, Syrah, Merlot, Chardonnay), one table grape cultivar (*V. vinifera*, cv. Thomson Seedless) and one juice grape cultivar (*V. labruscana *'Concord'). Cambial scrapings of canes from a single Merlot grapevine were used for isolating genomic-length replicative-form double-stranded (ds) RNA essentially as described by Valverde *et al. *[44]. The integrity of dsRNA preparations were verified by resolving in 0.8% agarose gels and using dsRNA-enriched preparation from citrus (Sour Orange) infected with *Citrus trizteza virus *(CTV). Unless otherwise mentioned, the same dsRNA preparation was used for cloning and sequencing of the entire genome of GLRaV-3.

### cDNA synthesis, RT-PCR, cloning and sequence analyses

The denatured dsRNA served as a template for cDNA synthesis. The reaction was carried out in 25 μL reaction mixture in the presence of gene-specific complementary primer using Superscript III reverse transcriptase kit (Invitrogen Corp, Carlsbad, CA) by following the manufacturer's instructions. The cDNA preparation was used as a template for subsequent PCR amplification of different portions of the virus genome (Additional file 1, Figure S1) using primers listed in Additional file 1, Table S1). These primers were initially designed based on nucleotide sequence information available for New York isolate of GLRaV-3 (AF037268), [26]. Primers for subsequent experiments were designed based on the sequence obtained for GLRaV-3 isolate from Washington (accession no. GU983863). Unless stated otherwise, locations of all primers were indicated using the entire genome sequence of GLRaV-3 isolate from Washington. Each PCR reaction in 20 μL consisted of a final concentration of 2-4 ng of template cDNA, 0.3 μMoles each of sense and antisense primers, 0.2 mM of each dNTP, 1X concentration of reaction buffer consisting 1 mM Mg^2+ ^and 0.025 U of high fidelity Takara Prime Star DNA polymerase (Takara Bio Inc., Japan). The temperature profile for the amplification included denaturation at 94°C for 30 sec followed by 35 cycles of denaturation at 98°C for 10 sec, annealing at 60°C for 5 sec and extension at 72°C for 60 sec per 1 kb size of amplicon and a final extension at 72°C for 10 minutes.

The amplicons were cloned in pUC-119 based vector and two independent clones specific to each amplicon were sequenced in both orientations at the Molecular Biology Core Instrumentation Facility, Washington State University, Pullman, WA. Wherever necessary, additional clones were sequenced to resolve nucleotide sequence ambiguities. Nucleotide and predicted amino acid sequences were analyzed using Vector NTI Advance11 software (Invitrogen Corp, Carlsbad, CA).

### Rapid amplification of cDNA ends of GLRaV-3

The exact 5' end sequence of GLRaV-3 was determined using a commercially available 5'RACE system for rapid amplification of cDNA ends kit (Version 2.0, Invitrogen, Carlsbad, CA). Total RNA was isolated from bark scrapings of canes collected from GLRaV-3-infected Merlot vines based on a protocol reported by Tattersall *et al.*[45]. First-strand cDNA was synthesized using the gene-specific primer AR (5'-CATTAAGGGCCCTGTTAAAC-3', complementary to nucleotides 833 to 852 of the Washington isolate, GU983863). The purified first-strand cDNA was 'dC' tailed and the following primer combinations were used to amplify virus-specific DNA fragments: virus-specific primer M1012 (5'-AAGTCCGACAACTTCACGTTCCCT-3', complementary to nucleotides 860 to 883 in GU983863) and the abridged anchor primer (AAP) supplied with the kit and virus-specific primer M1013 (5'-AAGTTGAGGTCCTTGCCTCCATCAAG-3', complementary to nucleotides 1034 to 1059 in GU983863) and the AAP supplied with the kit. The temperature profile for the amplification of DNA included denaturation at 94°C for 3 min followed by 35 cycles of denaturation at 94°C for 10 sec, annealing at 56°C for 30 sec and extension at 72°C for 60 sec and a final extension at 72°C for 5 min.

For verification of 3'end sequence of GLRaV-3, total RNA was poly-adenylated with Yeast Poly(A) polymerase (USB, Cleveland, OH) and used for cDNA synthesis using SuperScriptIII reverse transcriptase (Invitrogen, Carlsbad, CA). The first-strand cDNA was used as a template to amplify virus-specific DNA using an oligo dT primer M111 (5'-GGTCTCGAG(T)_18_-3') and a virus-specific forward primer GF (5'-ATTAGCATATGTAGAAAAGGGGAAG-3', nucleotides 18174 to 18198 in GU983863). The 5' and 3' RACE PCR products were cloned into pGEM-T vector (Promega, Madison, WI) and six independent clones specific to each PCR product were sequenced in both orientations. Sequences were analyzed as described above.

### Riboprobe preparation and Northern blot hybridization

Total RNA extracts prepared from cambial scrapings collected from GLRaV-3-infected Merlot grapevines were separated by electrophoresis in a formaldehyde denaturing 1.1% agarose gel in MOPS buffer, and transferred onto a nylon membrane using an electrotransfer unit (Hoefer Pharmacia Biotech, San fransisco, CA) as described by Lewandowski and Dawson [46]. Northern blot hybridization and probing of membranes for the presence of genomic RNA and subgenomic RNAs of GLRaV-3 with nonradioactive digoxigenin (DIG)-labeled riboprobes was carried out according to Tatineni *et al. *[33]. The probe for detecting all sgRNAs includes 17,899-18,498 nt at the 3' terminus of the Washington isolate of GLRaV-3 (Additional file 1, Table S5). DNA template for gene-specific riboprobe synthesis were amplified from virus-specific clones in pUC119 using the primers listed in Additional file 1, Table S5 and Table S6. T7 and SP6 polymerase promoter sequences were included in the forward and reverse primers, respectively, and the PCR amplified products were agarose-gel eluted and used as a template to generate positive-stranded RNA-specific probes by using T7- or SP6-RNA polymerase and nucleotides containing DIG-labeled UTP.

### Mapping the transcription start sites of GLRaV-3-sgRNAs

Total RNA isolated from cambial scrapings of GLRaV-3-infected Merlot grapevine was used to map the transcription start sites of four sgRNAs, namely, CP, p21, p20A and p20B. The gene specific primers (Additional file 1, Table S7) were used to synthesize the first-strand cDNAs. The first-strand cDNAs were column purified and 'dC' tailed at the 5' end using terminal deoxynucleotidyl transferase, and amplified by using 2.5 U of Taq DNA polymerase (New England Biolabs, Ipswich, MA) in a 50 μL reaction volume with an abridged anchor primer (supplied with kit) together with gene specific nested primer. The following conditions were used for PCR: 1 cycle at 94°C for 2 min, followed by 35 cycles at 94°C for 20 sec, 56°C for 20 sec and 72°C for 1 min, and one cycle at 72°C for 5 min. The PCR products were ligated into pGEM-T Easy vector (Promega, WI) and the inserts were sequenced at the DNA sequencing Core, Interdesciplinary Center for Biotechnology Research, University of Florida, Gainesville, FL.

### Sequence analysis

Sequences were edited and assembled using ContigExpress module in the VectorNTI sequence analysis software package (Invitrogen Corp, Carlsbad, CA). Nucleotide and amino acid sequence identity levels were calculated using Vector NTI Advance program (Invitrogen Corp. CA). Secondary structure analysis of the 5'NTR and 3'NTR sequences was carried out by using MFOLD software [30].

## Competing interests

The authors declare that they have no competing interests.

## Authors' contributions

RAN conceived and designed the study. SJ performed the research. SJ, SG, WOD and RAN analyzed the results and wrote the manuscript. All authors read and approved the final manuscript.

## Supplementary Material

Additional file 1 **Figure S1: Strategy for cloning GLRaV-3 genome, Table S1**: List of primers used to amplify the genome of GLRaV-3, **Table S2**: A comparison of nucleotide (nt) and amino acid (aa) sequence identities of different ORFs and 5' and 3' NTR of Washington isolate of GLRaV-3 with the corresponding sequences of virus isolates from New York, Chile and South Africa, **Table S3**: Characteristics of the four 3' co-terminal subgenomic RNAs of GLRaV-3, **Table S4**: A comparison of nucleotide and sequence identities between leader sequences of four subgenomic RNAs of Washington isolate of GLRaV-3 with corresponding sequences of virus isolates from New York (NY), Chile (Ch) and South Africa (SA), **Table S5**: List of primers used to amplify gene-specific fragments for preparing non-radioactive riboprobes, **Table S6**: List of primer combinations used to generate gene-specific riboprobes, Table S7: List of gene-specific primers used for mapping the 5' terminus of subgenomic RNAs. Click here for file
