# Supplementary material for: 3'-coterminal subgenomic RNAs and putative cis-acting elements of Grapevine leafroll-associated virus 3 reveals 'unique' features of gene expression strategy in the genus Ampelovirus
Source: Virol J. 2010 Aug 3;7:180. doi: 10.1186/1743-422X-7-180 (PMC2922190; doi:10.1186/1743-422X-7-180)
Supplement: Additional file 1 — Figure S1: Strategy for cloning GLRaV-3 genome, Table S1: List of primers used to amplify the genome of GLRaV-3, Table S2: A comparison of nucleotide (nt) and amino acid (aa) sequence identities of different ORFs and 5' and 3' NTR of Washington isolate of GLRaV-3 with the corresponding sequences of virus isolates from New York, Chile and South Africa, Table S3: Characteristics of the four 3' co-terminal subgenomic RNAs of GLRaV-3, Table S4: A comparison of nucleotide and sequence identities between leader sequences of four subgenomic RNAs of Washington isolate of GLRaV-3 with corresponding sequences of virus isolates from New York (NY), Chile (Ch) and South Africa (SA), Table S5: List of primers used to amplify gene-specific fragments for preparing non-radioactive riboprobes, Table S6: List of primer combinations used to generate gene-specific riboprobes, Table S7: List of gene-specific primers used for mapping the 5' terminus of subgenomic RNAs. [file 1743-422X-7-180-S1.DOC]

**Additional file 1**

**Legends**

**Figure S1**

**Title:** Strategy for cloning GLRaV-3 genome

**Description:** This figure shows the genomic map of GLRaV-3 (see Figure 1 for description of genome organization). The scale below the map indicates the size of GLRaV-3 genome. The location of primers used to amplify different portions of the virus genome is shown below the scale. Primer sequences used for RT-PCR amplification are listed in Additional file 1, Table S1.

**Table S1**
**Title:** List of primers used to amplify the genome of GLRaV-3
**Description:** This table shows list of primers used to amplify different portions of GLRaV-3 genome. The location of primer sequences in the virus genome are listed (positive sense as ‘+’ and complementary sense as ‘-’) based on the sequence of the Washington isolate of GLRaV-3 (Accession no. GU983863). The size of amplicons obtained with each primer pair is also listed.

**Table S2**
**Title:** A comparison of nucleotide (nt) and amino acid (aa) sequence identities of different ORFs and 5’ and 3’ NTR of Washington isolate of GLRaV-3 with the corresponding sequences of virus isolates from New York, Chile and South Africa.
**Description:** This table shows the size of different open reading frames (ORFs) and 3’ and 5’nontranslated regions (NTRs) of Washington isolate of GLRaV-3 (GU983863). The size of each ORF and both NTRs is indicated as the number of nucleotides. The number of amino acids for each ORF is indicated in parenthesis. Nucleotide sequence identity (amino acid sequence identity in parenthesis) for each ORF and 3’ and 5’NTRs between GLRaV-3 isolates from Washington, New York (AF037268), Chile (EU344893) and South Africa (EU259806) is also shown.

**Table S3**

**Title:** Characteristics of the four 3’ co-terminal subgenomic RNAs of GLRaV-3.

**Description:** This table shows the size of subgenomic (sg) RNA specific to coat protein (CP), p21, p20A and p20B of Washington isolate of GLRaV-3. The position of transcription start site and translation start codon for each sgRNA in the GLRaV-3 genome is listed. The size of the leader sequence for each sgRNA is also listed.

**Table S4**

**Title:** A comparison of nucleotide sequence identities between leader sequences of four subgenomic RNAs of Washington isolate of GLRaV-3 with corresponding sequences of virus isolates from New York (NY), Chile (Ch) and South Africa (SA).

**Description:** This table shows nucleotide sequence identity of subgenomic RNA leader sequences of the coat protein, p21, p20A and p20B between GLRaV-3 isolates from Washington, New York (NY), Chile (Ch) and South Africa (SA).

**Table S5**

**Title:** List of primers used to amplify gene-specific fragments for preparing non-radioactive riboprobes.
**Description:** This table shows a list of primers used to amplify different regions of GLRaV-3 genome for preparing non-radioactive probes used in Northern blot hybridization. The location of primer sequences (both positive and complementary sense) specific to CP, CPm, p21, p20A, p20B and 3’terminus in virus genome is indicated based on the genome sequence of the Washington isolate of GLRaV-3 (Accession no. GU983863). The sequence in bold represent SP6 RNA polymerase promoter and that in italics represent T7 RNA polymerase promoter.

**Table S6
Title:** List of primer combinations used to generate gene-specific riboprobes
**Description:** This table shows primer combinations used to generate RNA transcripts specific to CP, CPm, p21, p20A, p20B and 3’terminus of GLRaV-3. The size of gene-specific transcripts generated is also listed.

**Table S7**
**Title:** List of gene-specific primers used for mapping the 5’ terminus of subgenomic RNAs

**Description:** This table shows a list of complementary primers used in 5’RACE to amplify gene-specific fragments for determining the 5’ terminus of CP, p21, p20A and p20B subgenomic RNAs. The location of each primer in the virus genome is also listed.

**Figure S1:** Strategy for cloning GLRaV-3 genome.


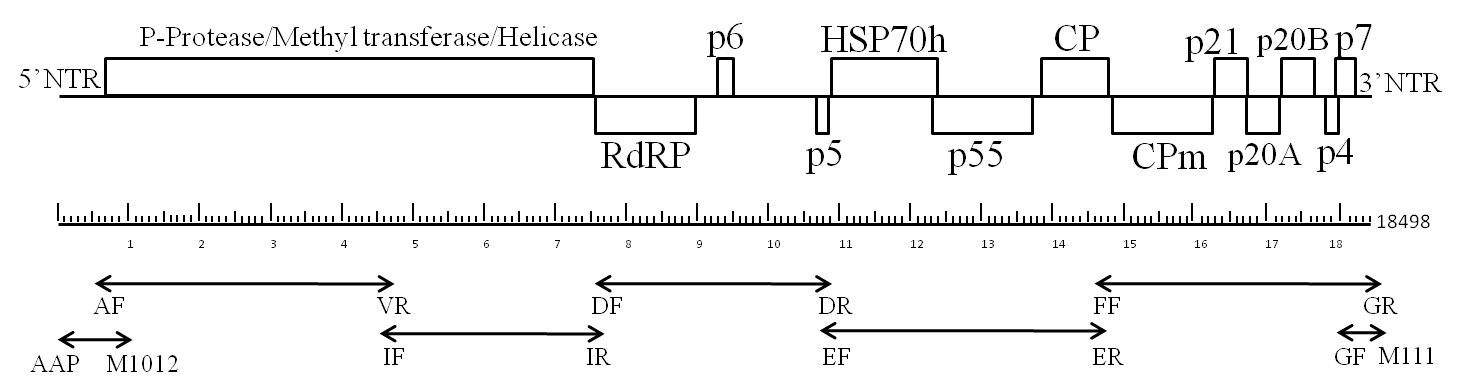


**Table S1:** List of primers used to amplify the genome of GLRaV-3

| Primer ID | Oligonucleotide sequence (5’ to 3’) | Location in GLRaV-3 genome (GU983863) | Polarity | Amplicon Size (bp) |
| --- | --- | --- | --- | --- |
| AAP | GGCCACGCGTCGACTAGTACGGGIIGGGIIGGGIIG | 5’RACE Kit* | (+) | 883 |
| M1012 | AAGTCCGACAACTTCACGTTCCCT | 860-883 | (-) |
| AF | CTAAGTAACACCTAGGAATTTCTACC | 580-605 | (+) | 4064 |
| VR | CATAGCTTGAGACACTAGAAGTGGATCCATCG | 4613-4644 | (-) |
| IF | TCCCGGTGACGATAACGATGGATCCACTTC | 4598-4627 | (+) | 2949 |
| IR | AGTAAGTCCTCGAGAAACCC | 7528-7547 | (-) |
| DF | GGGTTTCTCGAGGACTTACTC | 7528-7548 | (+) | 3470 |
| DR | AGGTGTCTGGTCCGGAAC | 10981-10998 | (-) |
| EF | TTTAGGTTCCGGACCAGAC | 10976-10994 | (+) | 3616 |
| ER | CGTTCATCACTAGTTTACCATTC | 14570-14592 | (-) |
| FF | GAATGGTAAACTAGTGATGAACG | 14570-14592 | (+) | 3928 |
| GR | GACCTAACTTATTGTCGATAAGTTAG | 18473-18498 | (-) |
| GF | ATTAGCATATGTAGAAAAGGAGAAG | 18174-18198 | (+) | 324 |
| M111 | GGTCTCGAG(T)_18_ | Oligo dT primer | (-) |

- Invitrogen, Carlsbad, CA (Cat # 18374-058)

**Table S2: A comparison of nucleotide (nt) and amino acid (aa) sequence identities of different ORFs and 5’ and 3’ NTR of Washington isolate of GLRaV-3 with the corresponding sequences of virus isolates from New York, Chile and South Africa.**

| Genomic region | Size  nt(aa) | Percent identity^1^ | | | |
| --- | --- | --- | --- | --- | --- |
|  |  | New York  (AF037268) | Chile  (EU344893) | | South Africa  (EU259806) |
| 5’NTR | 737 | 100* | 100* | 83 | |
| ORF1a (Methyl transferase/helicase) | 6714 (2237) | 96 (96) | 99 (98) | 91(93) | |
| ORF1b (RNA-dependent RNA polymerase) | 1617 (538) | 99 (100) | 100 (99) | 95 (98) | |
| ORF2 (6 kDa protein) | 156 (51) | 98 (96) | 99 (98) | 92 (84) | |
| ORF3 (5 kDa protein) | 138 (45) | 98 (100) | 99 (100) | 94 (98) | |
| ORF4 (Hsp70h-like protein) | 1650 (549) | 99 (99) | 99 (99) | 95 (98) | |
| ORF5 (p55 protein) | 1452 (483) | 100 (99) | 100 (100) | 93 (93) | |
| ORF6 (Coat protein) | 942 (313) | 99 (99) | 100 (100) | 93 (96) | |
| ORF7 (Coat protein duplicate) | 1434 (477) | 99 (97) | 99 (98) | 92 (90) | |
| ORF8 (21 kDa protein) | 558 (185) | 99 (99) | 99 (98) | 94 (97) | |
| ORF9 (19.6 kDa protein) | 534 (177) | 99 (99) | 99 (99) | 91 (88) | |
| ORF10 (19.7 kDa protein) | 540 (179) | 99 (97) | 99 (99) | 90 (88) | |
| ORF11 (4 kDa protein) | 111 (36) | 96 (92) | 98 (94) | 88 (83) | |
| ORF12 (7 kDa protein) | 183 (60) | 93 (92) | 99 (97) | 91 (88) | |
| 3’NTR | 277 | 97 | 100 | 97 | |

**^1^**Amino acid sequence in parenthesis

*Length of the 5’NTR in New York and Chile isolates is 158 nt and pairwise comparison was made with corresponding sequence only.

**Table S3:** Characteristics of the four 3’ co-terminal subgenomic RNAs of GLRaV-3.

| Subgenomic RNA | Position of transcription start site in virus genome | Position of start codon in virus genome | Size of leader sequence | Size of subgenomic RNA |
| --- | --- | --- | --- | --- |
| CP | 13800 | 13848 | 48 | 4699 |
| p21 | 16273 | 16296 | 23 | 2226 |
| p20A | 16755 | 16850 | 95 | 1744 |
| p20B | 17265 | 17390 | 125 | 1234 |

**Table S4**: A comparison of nucleotide sequence identities between leader sequences of four subgenomic RNAs of Washington isolate of GLRaV-3 with corresponding sequences of virus isolates from New York (NY), Chile (Ch) and South Africa (SA).

**NY Ch SA**

**CP:** 98 100 94

**p21:** 100 100 88

**p20A:** 100 98 93

**p20B:** 98 98 91

**Table S5:** List of primers used to amplify gene-specific fragments for non-radioactive riboprobes.

| Probe to | Primer ID | Oligonucleotide sequence (5’ to 3’) | Complementary site in GU983863 | Polarity |
| --- | --- | --- | --- | --- |
| CP | M893 | ATGGCATTTGAACTGAAATTAGGGCAG | 13848-13874 | (+) |
|  | M1151 | AGAC**ATTTAGGTGACACTATAG**CTCTTTGAACTCCGTCGAAGACG | 14398-14420 | (-) |
| CPm | M1031 | GTCTCCATGGGAGCTTATACACATGTAGAC | 14851-14875 | (+) |
|  | M1152 | AGAC**ATTTAGGTGACACTATAG**GTAGACCACTAACGTCCGTTTGC | 15331-15353 | (-) |
| p21 | M897 | ATGGAATTCAGACCAGTTTTAATTACAGTTCGCCG | 16296-16330 | (+) |
|  | M1164 | AGAC**ATTTAGGTGACACTATAG**CAATATCCCACACCACGCGCTATGGTC | 16728-16754 | (-) |
| p20A | M899 | ATGAAGTTGCTTTCGCTCCGCTATC | 16850-16874 | (+) |
|  | M1163 | AGAC**ATTTAGGTGACACTATAG**GCAACGTCGGATCCACAATCACCACT | 17228-17253 | (-) |
| p20B | M901 | ATGGACCTATCGTTTATTATTGTGCAGATCC | 17390-17420 | (+) |
|  | M1169 | AGAC**ATTTAGGTGACACTATAG**GTATGTCTGCTCCTTCAACTGCGGCCAGTCCG | 17803-17834 | (-) |
| 3’-terminus | M907 | *TAATACGACTCACTATAG*CTCTTGACGCTTTGTTGCGGAGCAC | 17899-17924 | (+) |
|  | M905 | **ATTTAGGTGACACTATAG**GACCTAACTTATTGTCGATAAGTTAGCC | 18471-18498 | (-) |

Sequence in bold = SP6 RNA polymerase promoter sequence

Sequence in italics = T7 RNA polymerase promoter sequence

**Table S6:** List of primer combinations used to generate gene-specific riboprobes

Size (nt/aa)

(AF037268)

(EU259806)

(EU344893)

5’UTR

737

100*

83

100*

ORF1a (Methyl transferase/helicase)

6714 (2237)

96 (96)

91(93)

99 (98)

ORF1b (RNA-dependent RNA polymerase)

1617 (538)

99 (100)

95 (98)

100 (99)

ORF2 (6 kDa protein)

156 (51)

98 (96)

92 (84)

99 (98)

ORF3 (5 kDa protein)

138 (45)

98 (100)

94 (98)

99 (100)

ORF4 (Hsp70-like protein)

1650 (549)

99 (99)

95 (98)

99 (99)

ORF5 (Hsp90-like protein)

1452 (483)

100 (99)

93 (93)

100 (100)

ORF6 (Coat protein)

942 (313)

99 (99)

93 (96)

100 (100)

ORF7 (Coat protein duplicate)

1434 (477)

99 (97)

92 (90)

99 (98)

ORF8 (21 kDa protein)

558 (185)

99 (99)

94 (97)

99 (98)

ORF9 (19.6 kDa protein)

534 (177)

99 (99)

91 (88)

99 (99)

ORF10 (19.7 kDa protein)

540 (179)

99 (97)

90 (88)

99 (99)

ORF11 (4 kDa protein)

111 (36)

96 (92)

88 (83)

98 (94)

ORF12 (7 kDa protein)

183 (60)

93 (92)

91 (88)

99 (97)

3’UTR

277

97

97

100

| Name of the RNA probe | Primer Pair | Transcript size (nt) |
| --- | --- | --- |
| CP specific probe | M893 & M1151 | 573 |
| CPm specific probe | M1031 & M1152 | 503 |
| p21 specific probe | M897 & M1164 | 459 |
| p20A specific probe | M899 & M1163 | 404 |
| p20B specific probe | M901 & M1169 | 445 |
| 3’-terminus probe | M907 & M905 | 600 |

**Table S7:** List of gene-specific primers used for mapping the 5’ terminus of subgenomic RNAs

| ORF | Primer ID | Oligonucleotide sequence (5’ to 3’) | Complementary site in GU983863 | Polarity |
| --- | --- | --- | --- | --- |
| CP | M917 | CCTTGTGCCGCATCCCCCACTCTAACTCTC | 13904-13933 | (-) |
| p21 | M923 | GTGTCGGTGTCTCGAAACGACTTTACCGCGCAG | 16403-16435 | (-) |
| p20A | M925 | GAGGCGTTGTAATAGTTTATAAGCGCCTCC | 16936-16965 | (-) |
| p20B | M927 | GCGGATCGTTTATCGCTGCCCAGCGCGTCG | 17493-17522 | (-) |
